# Post-Translational Deimination of Immunological and Metabolic Protein Markers in Plasma and Extracellular Vesicles of Naked Mole-Rat (*Heterocephalus glaber*)

**DOI:** 10.3390/ijms20215378

**Published:** 2019-10-29

**Authors:** Matthew E. Pamenter, Pinar Uysal-Onganer, Kenny W. Huynh, Igor Kraev, Sigrun Lange

**Affiliations:** 1 Department of Biology, University of Ottawa, Ottawa, ON K1N 6N5, Canada; mpamenter@uottawa.ca (M.E.P.); khuyn034@uottawa.ca (K.W.H.); 2 Brain and Mind Research Institute, University of Ottawa, Ottawa, ON K1H 8M5, Canada; 3 Cancer Research Group, School of Life Sciences, College of Liberal Arts and Sciences, University of Westminster, London W1W 6 UW, UK; P.onganer@westminster.ac.uk; 4 Electron Microscopy Suite, Faculty of Science, Technology, Engineering and Mathematics, Open University, Walton Hall, Milton Keynes MK7 6AA, UK; igor.kraev@open.ac.uk; 5 Tissue Architecture and Regeneration Research Group, School of Life Sciences, College of Liberal Arts and Sciences, University of Westminster, London W1W 6 UW, UK

**Keywords:** peptidylarginine deiminases (PADs), protein deimination, naked mole-rat (*Heterocephalus glaber*), extracellular vesicles (EVs), immunity, metabolism, microRNA (miR21; miR155; miR210)

## Abstract

Naked mole-rats are long-lived animals that show unusual resistance to hypoxia, cancer and ageing. Protein deimination is an irreversible post-translational modification caused by the peptidylarginine deiminase (PAD) family of enzymes, which convert arginine into citrulline in target proteins. Protein deimination can cause structural and functional protein changes, facilitating protein moonlighting, but also leading to neo-epitope generation and effects on gene regulation. Furthermore, PADs have been found to regulate cellular release of extracellular vesicles (EVs), which are lipid-vesicles released from cells as part of cellular communication. EVs carry protein and genetic cargo and are indicative biomarkers that can be isolated from most body fluids. This study was aimed at profiling deiminated proteins in plasma and EVs of naked mole-rat. Key immune and metabolic proteins were identified to be post-translationally deiminated, with 65 proteins specific for plasma, while 42 proteins were identified to be deiminated in EVs only. Using protein-protein interaction network analysis, deiminated plasma proteins were found to belong to KEEG (Kyoto Encyclopedia of Genes and Genomes) pathways of immunity, infection, cholesterol and drug metabolism, while deiminated proteins in EVs were also linked to KEEG pathways of HIF-1 signalling and glycolysis. The mole-rat EV profiles showed a poly-dispersed population of 50–300 nm, similar to observations of human plasma. Furthermore, the EVs were assessed for three key microRNAs involved in cancer, inflammation and hypoxia. The identification of post-translational deimination of critical immunological and metabolic markers contributes to the current understanding of protein moonlighting functions, via post-translational changes, in the longevity and cancer resistance of naked mole-rats.

## 1. Introduction

Peptidylarginine deiminases (PADs) are phylogenetically conserved calcium-dependent enzymes that post-translationally convert arginine into citrulline in target proteins in an irreversible manner. This can cause structural changes in target proteins and affect protein function, gene regulation and generation of neoepitopes [1,2,3,4,5,6]. Such post-translational changes in proteins may also allow for protein moonlighting, an evolutionary acquired phenomenon facilitating proteins to exhibit several physiologically relevant functions from within one polypeptide chain [7,8].

PADs and associated protein deimination are crucial players in cancer, autoimmune and neurodegenerative diseases [4,5,6,9], with recent indications also for PAD-mediated mechanisms in ageing [10]. Furthermore, critical roles have been identified for PADs and PAD-mediated protein deimination in response to hypoxia and in CNS regeneration [11,12,13,14,15] as well as in tissue remodelling and immunity [16,17,18]. Importantly, PADs have been found to have key roles in the regulation of extracellular vesicle (EV) release [19,20,21,22]. EVs are found in most body fluids and participate in cellular communication via transfer of cargo proteins and genetic material [6,23,24,25,26]. EVs isolated from serum, plasma and other body fluids can therefore be useful health biomarkers [27,28]. Hitherto, work on EVs has mainly been in the context of human pathologies and recent comparative immunology studies on EVs and EV cargo have been performed [29], including the assessment of deiminated EV protein cargo [30,31,32].

PADs have been identified throughout phylogeny from bacteria to mammals, with five tissue specific PAD isozymes in mammals, three in chicken, one in bony and cartilaginous fish [1,16,17,31,33] and PAD homologues in parasites [34], fungi [35] and bacteria [22]. While five PAD isozymes have been described in the naked mole-rat (PADI1, Gene ID: 101722077; PADI2, Gene ID: 101721485; PADI3, Gene ID: 101722435; PADI4, Gene ID: 101722785; PADI6, Gene ID: 101723122), no studies have been carried out on their deiminated protein products or the putative physiological relevance of such post-translational deimination in the physiology of the naked mole-rat.

The naked mole-rat is a burrowing rodent and eusocial mammal, and the only species in the genus *Heterochephalus* of the family *Heterochepalidae* [36,37]. They have a set of highly unusual physical traits, many of which are thought to derive from their highly-social and putatively hypoxic and hypercapnic subterranean lifestyle. For example, naked mole-rats are among the most hypoxia-tolerant mammal presently identified and tolerate minutes of anoxia, hours at 3% O_2_, and days to weeks at 8% O_2_ [38,39,40,41]. The key to tolerating prolonged hypoxia is to match metabolic demand to reduced energy (O_2_) supply [42,43,44,45], and in acute severe hypoxia (3% O_2_), the metabolic rate of adult naked mole-rats decreases up to 85% [40]. However, naked mole-rats remain conscious and active, albeit to a reduced degree [46,47,48]. These findings indicate that naked mole-rats are capable of significant metabolic plasticity within their natural environment. Conversely, naked mole-rats are largely non-responsive to hypercapnia and associated acidity-related pain responses are largely absent [49,50]. Naked mole-rats also have numerous adaptations that are not as obviously linked to their natural habitat, including a remarkable resistance to cancer [51,52], they are the only mammalian thermo-conformer and almost entirely ectothermic for regulation of body temperature [53,54] and they have remarkable longevity [55,56,57,58]. These traits make the naked mole-rat an important animal model for a range of human diseases and for furthering understanding of pathways underlying cancer resistance and longevity [59,60,61]. However, little is known about the immune system of naked mole-rats. As PAD-mediated pathways and EVs are increasingly recognized as key players in immune responses and metabolism, and related to a range of human inflammatory pathologies and cancer, a study on these parameters in mole-rat is warranted.

In the current study, plasma and plasma-derived EVs were profiled in naked mole-rats and assessed for deiminated protein profiles as well as three key microRNAs (miRs) related to inflammation and hypoxic resistance. For the first time we report on post-translational deimination of key immune and metabolic proteins in naked mole-rat and species-specific EV profiles.

## 2. Results

### 2.1. PAD Homologues in Naked Mole-Rat Plasma

**Figure 1:**
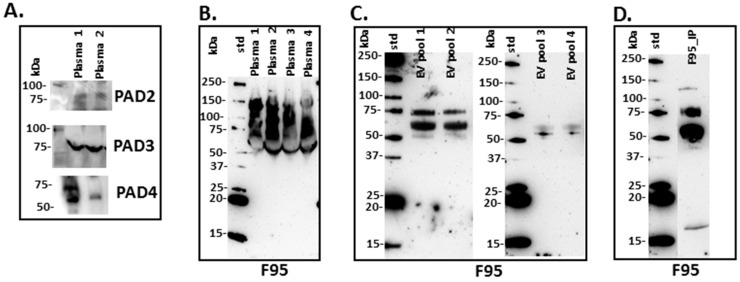
Peptidylarginine deiminases (PADs) and deiminated proteins in naked mole-rat plasma and plasma-extracellular vesicles (EVs). (**A**) PAD positive bands were identified at the expected size of approximately 70–75 kDa using the human PAD2, PAD3 and PAD4 specific antibodies in naked mole-rat plasma. (**B**) Total deiminated proteins were identified in naked mole-rat plasma (*n* = 4) using the F95 pan-deimination specific antibody. (**C**) Total deiminated proteins were identified in naked mole-rat plasma-EVs using the F95 pan-deimination specific antibody (EV pools from plasma of 4 individuals are shown, respectively). (**D**) The F95-enriched IP fraction from mole-rat plasma (from a pool of 5 individual mole-rat plasma; F95_IP) is shown. The molecular weight marker is indicated next to each blot.

Using PAD-isozyme specific antibodies, generated against human PADs, positive bands were observed by Western blotting and indicated PAD homologue proteins in mole-rat plasma at the expected size of approximately 70–75 kDa for PAD2, PAD3 and PAD4 (Figure 1A).

### 2.2. Deiminated Protein Profiles of Naked Mole-Rat Plasma and Plasma-Derived EVs

**Figure 2:**
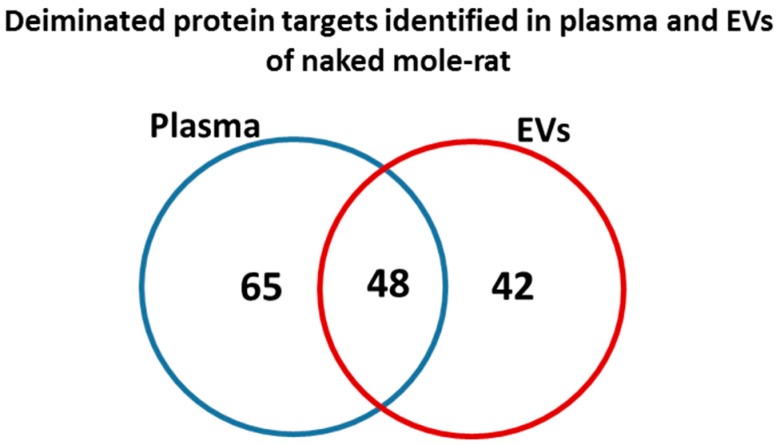
Deiminated proteins identified in naked mole-rat plasma and plasma-EVs. Species specific hits identified for deiminated proteins in naked mole-rat plasma and EVs showed 112 total proteins identified in plasma and 80 in EVs, respectively. Of these, 48 protein hits were overlapping, while 64 proteins were specific for whole plasma and 32 for plasma-EVs only, respectively.

**Figure 3:**
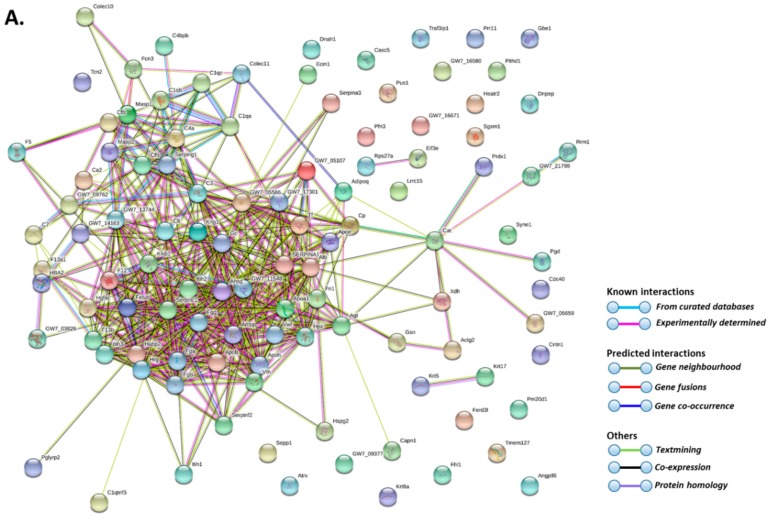

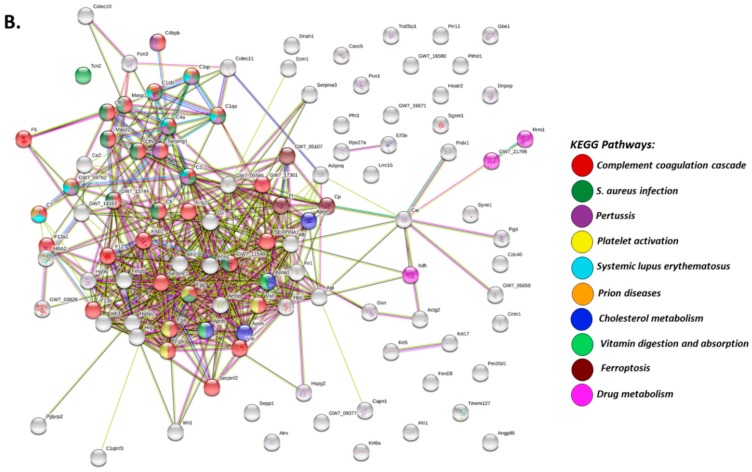
Protein-protein interaction networks of deiminated proteins identified in naked mole-rat plasma. Reconstruction of protein-protein interactions based on known and predicted interactions using STRING (Search Tool for the Retrieval of Interacting Genes/Proteins) analysis. (**A**) Coloured nodes represent query proteins and first shell of interactors. (**B**) KEGG (Kyoto Encyclopedia of Genes and Genomes) pathways relating to the identified proteins and reported in STRING are highlighted as follows: red = complement and coagulation cascade; dark green = *Staphylococcus aureus* infection; purple = pertussis; yellow = platelet activation; light blue = systemic lupus erythematosus (SLE); orange = prion diseases; dark blue = cholesterol metabolism; light green = vitamin digestion and absorption; dark red = ferroptosis; pink = drug metabolism. Coloured nodes represent query proteins and first shell of interactors; white nodes are second shell of interactors. Coloured lines indicate whether protein interactions are identified via known interactions (curated databases, experimentally determined), predicted interactions (gene neighbourhood, gene fusion, gene co-occurrence) or via text mining, co-expression or protein homology (see the colour key for connective lines included in the figure).

**Figure 4:**
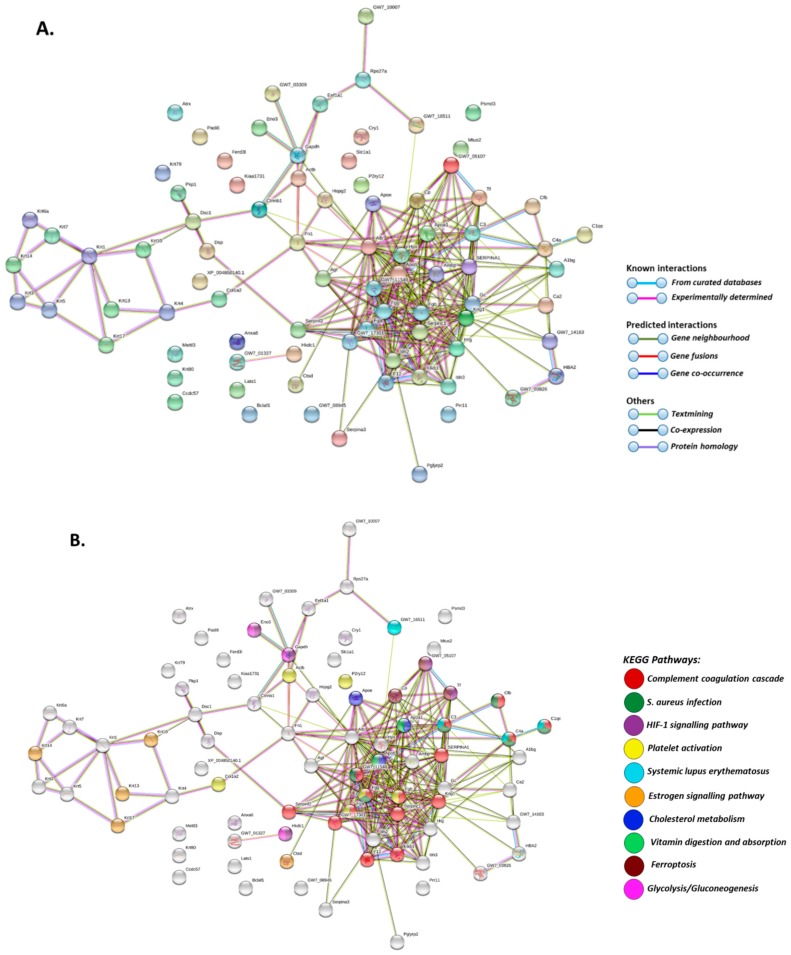
Protein-protein interaction networks of deiminated proteins identified in plasma-EVs of naked mole-rat. Reconstruction of protein-protein interactions based on known and predicted interactions using STRING analysis. (**A**) Coloured nodes represent query proteins and first shell of interactors. (**B**) KEGG pathways relating to the identified proteins and reported in STRING are highlighted as follows: red = complement and coagulation cascade; dark green = *Staphylococcus aureus* infection; purple = HIF-signalling pathway; yellow = platelet activation; light blue = systemic lupus erythematosus (SLE); orange = oestrogen signalling pathway; dark blue = cholesterol metabolism; light green = vitamin digestion and absorption; pink = glycolysis/gluconeogenesis. Coloured nodes represent query proteins and first shell of interactors, white nodes are second shell of interactors. Coloured lines indicate whether protein interactions are identified via known interactions (curated databases, experimentally determined), predicted interactions (gene neighbourhood, gene fusion, gene co-occurrence) or via text mining, co-expression or protein homology (see the colour key for connective lines included in the figure).

Total deiminated proteins were detected by Western blotting with the pan-deimination F95 antibody in mole-rat plasma and plasma-derived EVs, revealing a range of proteins mainly between 50–150 kDa (Figure 1B). The mono-specific F95 antibody was used in this study for the identification of deiminated proteins, as it has been developed against a deca-citrullinated peptide and is predicted to react with all deiminated/citrullinated proteins based on 100% sequence homology (MABN328 Merck), and it has been used to identify deiminated proteins in human and animals from diverse taxa [11,12,16,18,19,21,22,31]. Deiminated proteins in mole-rat were also detected in the plasma-derived EVs, mainly in the size range of 20–100 kDa (Figure 1C). Deiminated protein candidates in plasma and EVs were further identified by F95 enrichment (see F95 enriched fraction from plasma assessed by Western blotting, Figure 1D) and LC-MS/MS analysis (Table 1 and Table 2; Supplementary Tables S1 and S2). In plasma, 112 species-specific protein hits were identified (Table 1 and Supplementary Table S1) while in EVs, 80 protein hits were identified (Table 2 and Supplementary Table S2). Overall, 48 proteins overlapped between plasma and plasma-derived EVs, while 65 proteins were specific for whole plasma only and 42 proteins for EVs only (Figure 2). The protein lists for deiminated proteins identified in naked mole-rat plasma and plasma-EVs were submitted to STRING (Search Tool for the Retrieval of Interacting Genes/Proteins) analysis (https://string-db.org/) to predict putative protein-protein interaction networks (Figure 3 and Figure 4).

### 2.3. Characterisation of Extracellular Vesicles in Naked Mole-Rat Plasma

**Figure 5:**
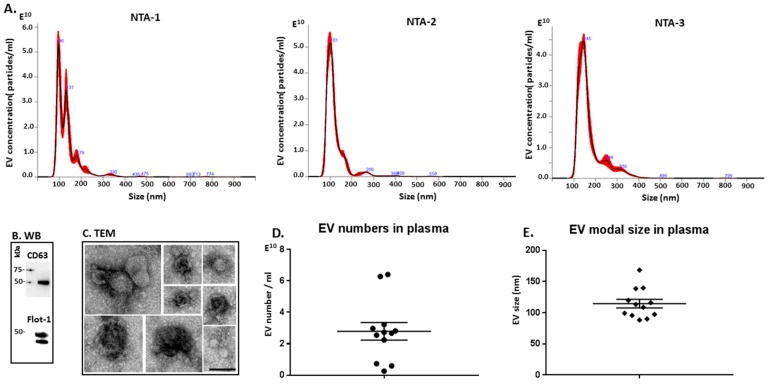
Extracellular vesicle profiling in naked mole-rat plasma. (**A**) Nanoparticle tracking analysis (NTA) shows a size distribution of EVs from naked mole-rat in the range of mainly 50 to 300 nm, with representative NTA profiles of EVs from 3 different animals (NTA-1, NTA-2, NTA-3). (**B**) Western blotting analysis confirms that naked mole-rat EVs are positive for the phylogenetically conserved EV-specific markers CD63 and Flot-1. (**C**) Transmission electron microscopy (TEM) analysis of naked mole-rat plasma-derived EVs shows typical EV morphology; a composite figure is shown and the scale bar (100 nm) applies for all images in the panel. (**D**) EV yield in plasma of 12 individual naked mole-rats is shown. (**E**) EV modal size in plasma of 12 individual naked mole-rats is presented.

EVs isolated from naked mole-rat plasma were characterised for size distribution using nanoparticle tracking analysis (NTA; Figure 5A), by Western blotting using EV-specific markers (Figure 5B) and by morphological analysis using transmission electron microscopy (TEM; Figure 5C). A poly-dispersed population of EVs mainly in the size range of 50 to 300 nm was observed, with some variations between individual animals (Figure 5A), and also with respect to EV yield from plasma (Figure 5D) and modal EV size (Figure 5E). Overall, the main EV peaks were detected at approximately 100–140 nm (Figure 5A,E), with the modal size of EVs falling mainly in the range of 90–115 nm, although this varied somewhat between animals and some outliers were detected (Figure 5E). Western blotting confirmed that the naked mole-rat EVs were positive for the EV-specific markers CD63 and Flot-1 (Figure 5B). Typical EV morphology was confirmed by TEM (Figure 5C).

### 2.4. MicroRNA Analysis of Naked Mole-Rat EVs

**Figure 6:**
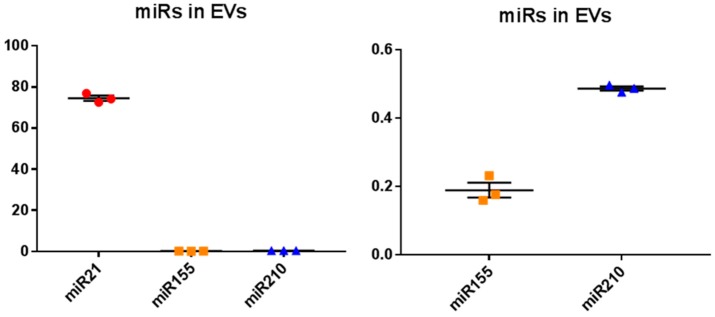
MicroRNA (miR) analysis of three key miRs related to cancer, inflammation, metabolism and hypoxia, in naked mole-rat plasma-derived EVs. (**A**). The onco-related miR21 showed the highest relative expression of the three miRs tested, being 394-fold higher than miR155 and 153-fold higher than miR210, respectively (*p* < 0.0001; *n* = 3). (**B**). The relative expression of the hypoxia and metabolic related miR210 was 2.6–fold higher than the inflammatory miR155 (*p* = 0.0002; *n* = 3); (miR21, miR155 and miR210 expression is represented as red circles, orange squares and blue triangles, respectively).

EVs isolated from naked mole-rat plasma were assessed for the relative expression of the immune- and cancer-related miR21, the inflammatory-related miR155 and the metabolic- and hypoxia-related miR210. The highest relative miR levels of the three miRs tested, were observed for miR21 in naked mole-rat plasma-EVs, 394-fold higher than for miR155 and 153-fold higher than for miR210, respectively (Figure 6A). The relative expression of the hypoxia- and metabolic-related miR210 was 2.6–fold higher than the inflammatory miR155 (Figure 6B), which overall showed comparatively the lowest relative levels of expression of the three miRs tested (Figure 6A,B).

## 3. Discussion

For the first time, post-translationally deiminated proteins are described in naked mole-rat (*Heterocephalus glaber*) plasma, unravelling novel aspects of post-translational deimination of key proteins involved in immune defences and metabolism. PAD homologues were identified in naked mole-rat plasma by Western blotting via cross reaction with human PAD2, which is the phylogenetically most conserved form of PAD [1,16,31]. PAD-positive protein bands were also observed for PAD3 and PAD4, via cross-reaction with PAD isozyme-specific human antibodies, at an expected size of approximately 70 kDa, similar to that reported for other mammalian PADs. Such protein detection corresponds with PAD isozymes that have been identified in the naked mole-rat genome (PADI1, Gene ID: 101722077; PADI2, Gene ID: 101721485; PADI3, Gene ID: 101722435; PADI4, Gene ID: 101722785; PADI6, Gene ID: 101723122). Deiminated proteins were detected and identified both in whole plasma and in plasma-derived EVs. The identity of specific deimination protein candidates in plasma and plasma-EVs was assessed using F95-enrichment in tandem with LC-MS/MS analysis. The F95 mono-specific antibody used in the current study was developed against a deca-citrullinated peptide and has been verified to detect deiminated/citrullinated proteins in diverse taxa [11,12,16,18,19,21,22,31]. In the naked mole-rat, species-specific protein hits revealed 48 common deiminated proteins in plasma and EVs by F95-enrichment, which included some key immune and metabolic related proteins, while 65 deiminated protein hits were specific for plasma and 42 deiminated protein hits specific for EVs only.

As assessed by STRING analysis, the PPI enrichment *p*-value for deiminated proteins identified in naked mole-rat plasma and as cargo in plasma-derived EVs was found to be <1.0 × 10^−16^ for both. Such an enrichment value indicates that the identified network of proteins has significantly more interactions than expected. Therefore, these deiminated proteins have more interactions among themselves than would be expected for a random set of proteins of similar size, drawn from the genome. Such an enrichment indicates that the proteins, as a group, are at least partially biologically connected. Deiminated target proteins identified in whole naked mole-rat plasma belonged to the following KEGG (Kyoto Encyclopedia of Genes and Genomes) pathways: complement coagulation cascade, platelet activation, cholesterol metabolism, vitamin digestion and adsorption, drug metabolism as well as bacterial infection (*Staphylococcus aureus* and pertussis), autoimmunity (systemic lupus erythematosus) and prion diseases (Figure 3B). Deiminated target proteins identified in EVs of naked mole-rat plasma belonged to KEGG pathways of the complement coagulation cascade, platelet activation, HIF- (hypoxia-inducible factor) signalling pathway, glycolysis/gluconeogenesis, cholesterol metabolism, vitamin digestion and adsorption, oestrogen signalling pathway, as well as bacterial infection (*Staphylococcus aureus*) and autoimmunity (systemic lupus erythematosus) (Figure 4B).

Of interest is that deiminated protein candidates involved in KEGG-pathways for HIF-1-signalling, the master regulator of oxygen homeostasis, seem enriched in the plasma-EVs, indicating a role for EV-mediated transport of such proteins in hypoxic signalling. It is worth noting that naked mole-rats have a high endogenous expression of HIF due to mutation in the VHL (Von Hippel-Lindau disease tumor suppressor) domain [62], which could possibly explain the elevated HIF-1 signalling related targets identified here. Furthermore, deiminated proteins identified in EVs were also enriched for glycolysis and gluconeogenesis KEGG pathways. Our findings indicate that protein deimination may play hitherto unidentified roles in the unusual hypoxia resistance and metabolism of the naked mole-rat, including via EV-transport in cellular communication, also under normal physiological conditions. In addition, the presence of deiminated histone H2B and H4 in EVs may be of some interest as histone deimination is well known to contribute to epigenetic regulation including in cancer [4,6], and the naked mole-rat has been found to have a particularly stable epigenome, which may contribute to the cancer resistance and longevity observed in these animals [63]. Furthermore, an abundance of deiminated complement components identified in both plasma and plasma-EVs may indicate roles for functional diversity of the complement system via post-translational deimination in the naked mole-rat. This may play various roles in naked mole-rat immune responses as recent studies have identified some unusual characteristics, including atypical immune surveillance and a greater reliance on myeloid-biased innate immunity [64]. Also noteworthy is the identification of deiminated adiponectin in naked mole-rat plasma identified here, as adiponectin in humans is the most abundant secreted adipokine with pleiotropic roles in metabolism [65,66], glucose regulation [67,68,69], longevity [70], regeneration and cancer [71,72,73]. Such deimination of adiponectin has not been studied and may add to some of its protein moonlighting function and be of relevance in the context of the unusual metabolism of the naked mole-rat.

As part of EV-mediated cellular communication in physiology and pathologies, the transport of microRNAs (miRs) is well acknowledged. There is increasing interest, reflected in a range of studies, in furthering our understanding of how such EV-mediated transport may play a part in physiological and pathophysiological processes. MiRs are highly conserved small non-coding RNAs that control gene expression and regulate biological processes by targeting messenger RNAs (mRNAs). MiRs can, for example, inhibit post-transcriptional translation of mRNA as well as enhance mRNA degradation [74]. Some expression profiling has been carried out in naked mole-rats, mainly at the transcriptome level [75,76], although no studies have assessed miRs in EVs of naked mole-rat plasma. This study focused on assessing three key miRs known to be involved in cancer, inflammation and hypoxia due to the unusual resistance of naked mole-rats to cancer, ageing and hypoxia. MiR21 is a main immunoregulatory and onco-related miR and is also associated with chronic diseases [77,78,79]. MiR21 is strongly conserved throughout evolution and while many experimentally verified targets of miR21 are tumour suppressors, miR21 is also linked to cardiac disease and oxidative stress [80]. Less is known about the physiological roles of miR21. In the current study, miR21 was found to be by far the highest miR expressed in EVs of naked mole-rat out of the three miRs tested. Roles for miR21 in immune responses of naked mole-rat have not been reported in detail and the expression of miR21 in EVs has not been assessed in naked mole-rat before.

In mammals, miR155 is known to be a major inflammatory related miR, linked to inflammatory and stress responses [81]. Here, miR155 was found to be the least expressed of the three miRs tested in naked mole-rat plasma-EVs, possibly indicating that this miR may be a contributing factor to the “anti-inflammatory” state of mole-rats, which may have some relation to their longevity and cancer resistance. MiR210 is known to be a major miR induced under hypoxia and has an important role in mitochondrial metabolism, DNA damage response, cell proliferation and apoptosis [74]. MiR210 has an important role in regulating mitochondrial metabolism [82] and cell glycolytic activity, as well as being linked to inflammation [83]. MiR210 has been identified as a regulator of the hypoxia pathway and was found to have pro-apoptotic functions under normoxic conditions, but anti-apoptotic effects under hypoxic conditions [84,85]. In the current study, miR210 was found to be more highly expressed in mole-rat plasma than the inflammatory miR155. As naked mole-rats are known to be hypoxia tolerant animals and to exhibit marked changes in their metabolic substrate use and metabolic demand in hypoxia [41,86], miR210 may have functional roles in metabolic control, possibly contributing to the well-known longevity of these animals. As this is the first study to assess the expression of these three onco-, inflammatory- and metabolic-related miRs in naked mole-rat plasma-EVs, it remains to be fully understood what specific functions the EV-mediated transport of these miRs play in the unusual physiology of naked mole-rats.

Here, for the first time, we report the protein deimination profiles of plasma and plasma-derived EVs in naked mole-rats. Post-translational deimination of major key immune and metabolic factors in naked mole-rats was identified and related to key KEGG pathways of inflammation, metabolism and oxygen transport. Our findings highlight novel aspects of protein moonlighting via post-translational deimination, including via EV-mediated transport. Research on EVs is a relatively new field in comparative animal models, and to our knowledge this is the first characterisation of EVs and associated protein and selected miR cargo markers in naked mole-rats. Furthermore, as PADs have been found to play major roles in the regulation of EV release [19,20,21,22,34], their contribution to EV-mediated cell communication in response to physiological and pathophysiological changes in naked mole-rats remains to be further investigated. Findings in long-lived mammals that display cancer resistance, including naked mole-rats, may be of considerable translational value for furthering our understanding of the mechanisms underlying cancer resistance for improved development of human cancer therapies [59].

In continuation of the current study, the assessment of changes in deiminated proteins and EV profiles, including protein and genetic EV-cargo, may be of great interest in studies using this unique animal model to further understanding of the hitherto novel and understudied mechanisms involved in cancer and ageing.

## 4. Materials and Methods

### 4.1. Sampling of Naked Mole-Rat Plasma

Naked mole-rats were group-housed in interconnected multi-cage systems at 30 °C and 21% O_2_ in 50% humidity with a 12L:12D light cycle. Animals were fed fresh tubers, vegetables, fruit and Pronutro cereal supplement ad libitum. Animals were not fasted prior to experimental trials. All experimental procedures were approved by the University of Ottawa Animal Care Committee in accordance with the Animals for Research Act and by the Canadian Council on Animal Care (protocol # 2535). Non-breeding (subordinate) naked mole-rats do not undergo sexual development or express sexual hormones and thus we did not take sex into consideration when evaluating our results [87]. Blood was collected from 12 adult (~ 1–2 years old) subordinate naked mole-rats following live cervical dislocation and rapid decapitation. Blood was collected in Eppendorf tubes pre-coated with a 10% EDTA (ethylenediaminetetraacetic acid) solution. Plasma was isolated by centrifugation at 5000× *g* for 5 min. The isolated plasma was aliquoted and immediately frozen at −80 °C until further use.

### 4.2. Extracellular Vesicle Isolation and Nanoparticle Tracking Analysis (NTA)

EVs were isolated by step-wise centrifugation according to our established protocols using ultracentrifugation and the recommendations of MISEV2018 (the minimal information for studies of extracellular vesicles 2018; [88]). Mole-rat plasma were diluted 1:4 in ultra-filtered (using a 0.22 μm filter) Dulbecco’s PBS (100 μL plasma added to 400 μL DPBS) and then centrifuged at 4000× *g* for 30 min at 4 °C for removal of aggregates and apoptotic bodies. The supernatants were collected and centrifuged further at 100,000× *g* for 1 h at 4 °C. The EV-enriched pellets were washed in 1 mL DPBS and ultra-centrifuged at 100,000× *g* for 1 h at 4 °C. The final EV pellets were resuspended in 100 µL DPBS and frozen at −80 °C until further use. For NTA, based on Brownian motion of particles in suspension, the EV pellets were diluted 1/100 in DPBS and applied to the NanoSight NS300 system (Malvern Panalytical Ltd., Malvern, UK) in conjunction with a syringe pump to ensure continuous flow of the sample. Five 60 sec videos were recorded for each sample, with approximately 40–60 particles per frame, and the replicate histograms generated were averaged.

### 4.3. Transmission Electron Microscopy (TEM)

EVs were isolated from individual plasma as described above. For TEM, the EV pellets were fixed with 2.5% glutaraldehyde in 100 mM sodium cacodylate buffer (pH 7.0) for 1 h at 4 °C. The EVs were then resuspended in 100 mM sodium cacodylate buffer (pH 7.0), placed on to a grid with a glow discharged carbon support film and stained with 2% aqueous Uranyl Acetate (Sigma-Aldrich, Gillingham, UK). Individual EVs were imaged by TEM using a Morada CCD camera (EMSIS GmbH, Münster, Germany) and processed via iTEM (EMSIS).

### 4.4. Western Blotting Analysis

Mole-rat plasma and plasma-EVs (an EV pellet derived from 100 µL plasma, reconstituted in 100 µL PBS) were diluted 1:1 in 2 × Laemmli sample buffer, boiled for 5 min at 100 °C and separated by SDS-PAGE on 4–20% TGX gels (BioRad, Watford, UK). Approximately 5 μg protein was loaded per lane. Following SDS-PAGE, proteins were transferred to nitrocellulose membranes using semi-dry Western blotting; even transfer was assessed by PonceauS (Sigma-Aldrich, Gillingham, UK) staining. The membranes were blocked in 5% BSA in TBS-T for 1 h at room temperature (RT) and thereafter incubated with the following primary antibodies diluted in TBS-T: F95 pan-deimination antibody (MABN328, Merck, Watford, UK, 1/1000); PAD2 (ab50257, Abcam, Cambridge, UK, 1/1000); PAD3 (ab50246, Abcam, 1/1000); PAD4 (ab50247, Abcam, 1/1000); and two EV-specific markers: CD63 (ab216130, Abcam, 1/1000); Flot-1 (ab41927, Abcam, 1/2000). After primary antibody incubation overnight at 4 °C on a shaking platform, the membranes were washed for 3 × 10 min in TBS-T at RT and thereafter incubated with the appropriate HRP-conjugated secondary antibodies (anti-rabbit IgG BioRad or anti-mouse IgM BioRad, diluted 1/4000 in TBS-T) for 1 h, at RT. Membranes were washed for 5 × 10 min in TBS-T, followed by 1 × 10 min in TBS proteins bands were visualised using enhanced chemiluminescence (ECL, Amersham, Buckinghamshire, UK) and digital images were obtained using the UVP BioDoc-ITTM System (Thermo Fisher Scientific, Hemel Hempstead, UK).

### 4.5. Immunoprecipitation and Protein Identification

Deiminated proteins in plasma and in plasma-derived EVs were immunoprecipitated by enrichment with the F95 pan-deimination antibody (MABN328, Merck, Watford, UK), which has been developed against a deca-citrullinated peptide and specifically detects proteins modified by citrullination [89]. The mono-specific F95 antibody is predicted to react with all deiminated/citrullinated proteins based on 100% sequence homology and has for example been used to identify deiminated proteins in human, mouse, rat, chicken and teleost fish tissue [11,12,16,18,19,21,22,31,89]. The Catch and Release immunoprecipitation kit (Merck, Watford, UK) was used according to the manufacturer’s instructions. For F95 enrichment, plasma was pooled from 5 individual animals (5 × 20 μL), while for EVs, total protein was first extracted from the EV pellets derived from 100 μL plasma per animal, using 100 μL RIPA + buffer on ice for 2 h followed by centrifugation at 16,000× *g* for 30 min to collect the supernatant containing the proteins. The immunoprecipitation was carried out on a rotating platform overnight at 4 °C, and the F95 bound proteins were eluted using denaturing elution buffer according to the manufacturer’s instructions (Merck). The F95 enriched eluates were then either analysed by Western blotting or by liquid chromatography with tandem mass spectrometry (LC-MS/MS; Cambridge Proteomics, Cambridge, UK). Peak files obtained were submitted to Mascot (Matrix Science). An in-house database (Cambridge proteomics) for naked mole-rat was used for the identification of species-specific protein hits (CCP_Heterocephalus_glaber Heterocephalus_glaber_20190911; 21449 sequences; 10466552 residues).

### 4.6. MicroRNA Analysis

EV isolates from individual naked mole-rat plasma (from 100 μL plasma as before) were assessed for relative expression of 3 key microRNAs (miRs) related to oncogenic, inflammatory and metabolic activity. These selected miRs included two cancer and immune-related miRs, miR21 and miR155, and miR210 for hypoxia and metabolic activity. Total RNA was extracted from mole-rat plasma EVs (prepared as before) using Trizol (Sigma-Aldrich, Gillingham, UK). The purity and concentration of the isolated RNA were measured using the NanoDrop Spectrophotometer at 260 nm and 280 nm absorbance. The cDNA was produced using the qScript microRNA cDNA Synthesis Kit (Quantabio, Beverly, MA, USA) according to the manufacturer’s instructions and used to assess the expression of miR21, miR155 and miR210. Reference RNAs used for the normalization of miR expression levels were U6-snRNA and has-let-7a-5p. The PerfeCTa SYBR Green SuperMix (Quantabio, Beverly, MA, USA) was used together with MystiCq microRNA qPCR primers for the miR21 (hsa-miR-21-5p), mir155 (hsa-miR-155-5p) and miR210 (hsa-miR-210-5p). All miR primers were obtained from Sigma-Aldrich (UK). Thermocycling conditions were used as follows: denaturation at 95 °C for 2 min, followed by 40 cycles of 95 °C for 2 s, 60 °C for 15 s, and extension at 72 °C for 15 s. The 2^ΔΔCt^ method [90] was used for calculating relative miR expression levels and for normalisation. Each experiment was performed in 3 individuals, in triplicate.

### 4.7. Statistical Analysis

The histograms and graphs were prepared using the Nanosigh NS300 software (Malvern Panalytical Ltd., Malvern, UK) and GraphPad Prism version 7 (GraphPad Software, San Diego, CA, USA). Experiments were repeated in triplicate, histograms represent mean of data and standard error of mean (SEM) is indicated by the error bars. Significant differences were considered as *p* ≤ 0.05, following one-way ANOVA or Student’s t-test.

## 5. Conclusions

Here, for the first time, we report the protein deimination profiles of plasma and plasma-derived EVs in naked mole-rats. Post-translational deimination of major key immune and metabolic proteins in naked mole-rats was identified and related to key KEGG pathways of inflammation, metabolism and oxygen transport. Our findings highlight novel aspects of protein moonlighting via post-translational deimination, including via EV-mediated transport of such proteins in cellular communication. Three key microRNAs for oncogenic, inflammatory and metabolic/hypoxia function were also assessed in mole-rat plasma EVs. In continuation of the current study, the assessment of changes in deiminated proteins and EV profiles, including protein and microRNA EV-cargo may be of great interest in studies using this unique animal model to further understanding of hitherto novel and understudied mechanisms involved in cancer, inflammatory diseases and ageing.

## Figures and Tables

**Table 1:** Deiminated proteins identified by F95 enrichment in total plasma of naked mole-rat (*Heterocephalus glaber*). Deiminated proteins were isolated by immunoprecipitation using the pan-deimination F95 antibody. The F95 enriched eluate was analysed by LC-MS/MS and peak list files were submitted to mascot. Species-specific peptide sequence hits scoring with *H. glaber* are included and total score is shown. Protein hits identified in whole plasma only (but not in plasma EVs) are highlighted in pink. For full LC-MS/MS data analysis see Supplementary Table S1.

Protein Name	Symbol	Total Score (*p* < 0.05) ^#^
Apolipoprotein B-100	G5AZB7_HETGA	6077
Alpha-2-macroglobulin	G5BPM1_HETGA	4974
Complement C3	G5C0N5_HETGA	4300
Serotransferrin	G5BQA9_HETGA	4058
Serum albumin	G5B5P2_HETGA	3394
Fibronectin	G5BHR4_HETGA	3333
Fibrinogen beta chain	G5BML3_HETGA	2638
Kininogen-1	G5BT86_HETGA	2276
Fibrinogen alpha chain	G5BML2_HETGA	2094
Ceruloplasmin	G5BSL1_HETGA	2022
Histidine-rich glycoprotein	G5BT87_HETGA	1851
Complement C4-A	G5C3H6_HETGA	1759
Complement factor H	G5BM71_HETGA	1646
Plasminogen	G5BC53_HETGA	1548
Inter-alpha-trypsin inhibitor heavy chain H4	G5BUN4_HETGA	1455
Fibrinogen gamma chain	G5BML1_HETGA	1338
Complement factor B	G5C3H1_HETGA	1313
Plasma kallikrein	G5BNV2_HETGA	1167
Antithrombin-III	G5ARS6_HETGA	1093
Coagulation factor V	G5CB46_HETGA	1092
Complement C5	G5AXS5_HETGA	1002
Hemoglobin subunit beta	G5BS33_HETGA	941
Transcobalamin-2 isoform 1	G5AVP0_HETGA	913
Hemopexin	G5BBR0_HETGA	899
Coagulation factor XIII B chain	G5BM72_HETGA	821
Protein AMBP	G5B1Y4_HETGA	817
N-acetylmuramoyl-L-alanine amidase	G5BYP3_HETGA	812
Coagulation factor XII	G5BQ09_HETGA	802
Apolipoprotein A-I	APOA1_HETGA	798
Hemoglobin subunit alpha	G5BXY1_HETGA	784
Coagulation factor XIII A chain	G5BAS8_HETGA	779
Hemoglobin subunit beta	G5BYJ8_HETGA	759
Alpha-1-antiproteinase S	G5B496_HETGA	724
Inter-alpha-trypsin inhibitor heavy chain H3	G5BUN3_HETGA	682
Vitronectin	G5BVN8_HETGA	652
Complement factor I	G5AQM1_HETGA	647
Hemoglobin subunit epsilon-1	G5BS35_HETGA	642
Inter-alpha-trypsin inhibitor heavy chain H2	G5AXV8_HETGA	584
Apolipoprotein E	G5CBM7_HETGA	581
Inhibitor of carbonic anhydrase	G5BQB0_HETGA	537
Inter-alpha-trypsin inhibitor heavy chain H1	G5BUN2_HETGA	482
Four and a half LIM domains protein 1	G5CA61_HETGA	469
Haptoglobin	G5B5U6_HETGA	464
C4b-binding protein	G5BP10_HETGA	438
L-lactate dehydrogenase	G5AKA3_HETGA	437
Insulin-like growth factor-binding protein complex acid labile chain	G5BY64_HETGA	403
Catalase	G5AXV0_HETGA	382
Fetuin-B	G5BT88_HETGA	347
Alpha-2-HS-glycoprotein	G5BT89_HETGA	337
Selenoprotein P	G5APA7_HETGA	328
Vitamin D-binding protein	G5BE53_HETGA	317
Adiponectin	G5BT83_HETGA	283
von Willebrand factor	G5CAN6_HETGA	274
Beta-2-glycoprotein 1	G5BGY7_HETGA	268
Basement membrane-specific heparan sulfate proteoglycan core protein	G5BI06_HETGA	267
Gelsolin	G5AXS0_HETGA	259
Aspartyl aminopeptidase	G5AKJ4_HETGA	256
Complement C1q subcomponent subunit A	G5BHZ8_HETGA	246
Ficolin-3	G5AUT5_HETGA	241
Mannan-binding lectin serine protease 1	G5BTD5_HETGA	228
Alpha-1-antichymotrypsin	G5B491_HETGA	228
Carboxypeptidase N catalytic chain	G5AYP3_HETGA	210
Complement C1q subcomponent subunit C	G5BHZ7_HETGA	191
Mannan-binding lectin serine protease 2	G5C521_HETGA	188
Prothrombin	G5ATC4_HETGA	177
Complement C1q tumour necrosis factor-related protein 3	G5BQ97_HETGA0	177
Nucleoside diphosphate kinase	G5C4L3_HETGA	168
L-lactate dehydrogenase	G5BEG2_HETGA	167
Alpha-2-antiplasmin	G5BCV1_HETGA	164
Keratin, type II cytoskeletal 6B	G5ALS1_HETGA	152
Carbonic anhydrase 2	G5ATW7_HETGA	143
Proprotein convertase subtilisin/kexin type 9	G5APQ4_HETGA	137
Angiotensinogen	G5BQI5_HETGA	121
6-phosphogluconate dehydrogenase, decarboxylating	G5C530_HETGA	91
Nesprin-1	G5C0E1_HETGA	87
Complement C1q subcomponent subunit B	G5BHZ6_HETGA	87
Angiopoietin-related protein 6	G5B341_HETGA	85
Hepatocyte growth factor activator	G5BZF5_HETGA	84
Calpain-1 catalytic subunit	G5B6L3_HETGA	84
Sulfhydryl oxidase	G5AYL7_HETGA	80
Keratin, type II cytoskeletal 5	G5ALS3_HETGA	76
Profilin	G5BH50_HETGA	75
40S ribosomal protein S27a	G5B8W4_HETGA	68
Xanthine dehydrogenase/oxidase	G5B3Z0_HETGA	68
Plasma protease C1 inhibitor	G5BLJ5_HETGA	67
Collectin-11	G5C7L1_HETGA	66
Extracellular matrix protein 1	G5BH40_HETGA	64
Actin, gamma-enteric smooth muscle	G5AXH0_HETGA	61
Collectin-10	G5C9R8_HETGA	61
Keratin, type I cytoskeletal 17	G5B0M4_HETGA	58
Contactin-1	G5BGJ6_HETGA	55
Dynein heavy chain 1, axonemal	G5BUL8_HETGA	50
Leucine-rich repeat-containing protein 15	G5ALE6_HETGA	43
Transcriptional regulator ATRX	G5C0I5_HETGA	42
Transmembrane protein 127	G5BMW1_HETGA	41
TRAF3-interacting protein 1	G5BHH8_HETGA	41
Mannose-binding protein A	G5C4H7_HETGA	40
Hyaluronan-binding protein 2	G5BKD5_HETGA	39
Fer3-like protein	G5BZJ1_HETGA	38
Complement component C7	G5C4R4_HETGA	38
PITH domain-containing protein	G5BHY3_HETGA	38
1,4-alpha-glucan-branching enzyme	G5BA56_HETGA	37
tRNA pseudouridine synthase	G5BB54_HETGA	37
SRRM2-like protein	G5BG61_HETGA	34
Putative carboxypeptidase PM20D1	G5BX50_HETGA	34
Olfactory receptor	G5C741_HETGA	33
Protein CASC5	G5BLZ4_HETGA	33
Conserved oligomeric Golgi complex subunit 1	G5B5M3_HETGA	32
Small G protein signalling modulator 1	G5BGN5_HETGA	32
Eukaryotic translation initiation factor 3 subunit E	G5BD61_HETGA	31
HEAT repeat-containing protein 2	G5AZ15_HETGA	31
Proline-rich protein 11	G5AXY5_HETGA	31
Peroxiredoxin-1	G5ARW1_HETGA	30
Pre-mRNA-processing factor 17	G5B0Y2_HETGA	30
Ribonucleoside-diphosphate reductase	G5B9C2_HETGA	30

^#^ Ions score is −10 * Log(*p*), where *p* is the probability that the observed match is a random event. Individual ions scores > 30 indicate identity or extensive homology (*p* < 0.05). Protein scores are derived from ions scores as a non-probabilistic basis for ranking protein hits. Cut-off was set at Ions score 30. Protein hits identified in whole plasma only (but not in plasma EVs) are highlighted in pink.

**Table 2:** Deiminated proteins identified by F95 enrichment in plasma-derived EVs of naked mole-rat (*Heterocephalus glaber*). Deiminated proteins were isolated by immunoprecipitation using the pan-deimination F95 antibody. The F95 enriched eluate was analysed by LC-MS/MS and peak list files were submitted to mascot. Species-specific peptide sequence hits scoring with *H. glaber* are included and total score is shown. Protein hits identified in plasma-EVs only are highlighted in blue. For full LC-MS/MS data analysis see Supplementary Table S2.

Protein Name	Symbol	Total Score (*p* < 0.05) ^#^
Alpha-2-macroglobulin	G5BPM1_HETGA	2978
Serum albumin	G5B5P2_HETGA	2570
Serotransferrin	G5BQA9_HETGA	1976
Keratin, type II cytoskeletal 6B	G5ALS1_HETGA	1459
Complement C3	G5C0N5_HETGA	1440
Keratin, type II cytoskeletal 5	G5ALS3_HETGA	1211
Kininogen-1	G5BT86_HETGA	1196
Histidine-rich glycoprotein	G5BT87_HETGA	1186
Keratin, type I cytoskeletal 10	G5AX66_HETGA	1130
Keratin, type I cytoskeletal 14	G5B0M6_HETGA	1038
Keratin, type II cytoskeletal 1	G5ALS8_HETGA	1011
Fibrinogen beta chain	G5BML3_HETGA	974
Fibrinogen alpha chain	G5BML2_HETGA	956
Apolipoprotein B-100	G5AZB7_HETGA	834
Keratin, type I cytoskeletal 13	G5B0N0_HETGA	701
Keratin, type I cytoskeletal 17	G5B0M4_HETGA	673
Desmoplakin	G5BAT4_HETGA	670
Hemoglobin subunit alpha	G5BXY1_HETGA	591
Hemoglobin subunit beta	G5BS33_HETGA	581
Complement C4-A	G5C3H6_HETGA	571
Hemoglobin subunit beta	G5BYJ8_HETGA	568
Apolipoprotein A-I	APOA1_HETGA	499
Keratin, type II cytoskeletal 79	G5BJ37_HETGA	468
Actin, cytoplasmic 1	G5BI78_HETGA	444
Keratin, type II cytoskeletal 4	G5BJ36_HETGA	443
Fibrinogen gamma chain	G5BML1_HETGA	382
Ceruloplasmin	G5BSL1_HETGA	350
Apolipoprotein E	G5CBM7_HETGA	338
Inter-alpha-trypsin inhibitor heavy chain H4	G5BUN4_HETGA	332
Protein AMBP	G5B1Y4_HETGA	280
Inhibitor of carbonic anhydrase	G5BQB0_HETGA	244
Hemopexin	G5BBR0_HETGA	239
Histone H4	G5BKL3_HETGA	238
Plasminogen	G5BC53_HETGA	232
Alpha-1-antiproteinase S	G5B496_HETGA	217
Hemoglobin subunit epsilon-1	G5BS35_HETGA	213
Junction plakoglobin	G5B0M0_HETGA	202
N-acetylmuramoyl-L-alanine amidase	G5BYP3_HETGA	190
Inter-alpha-trypsin inhibitor heavy chain H3	G5BUN3_HETGA	190
Keratin, type II cytoskeletal 7	G5BL96_HETGA	182
Fibronectin	G5BHR4_HETGA	164
Glyceraldehyde-3-phosphate dehydrogenase	G5CAP7_HETGA	154
Coagulation factor XII	G5BQ09_HETGA	152
Antithrombin-III	G5ARS6_HETGA	150
Catenin beta-1	G5ALX2_HETGA	126
Plakophilin-1	G5B3A4_HETGA	120
Annexin	G5AWC0_HETGA	119
Keratin, type II cytoskeletal 3	G5ALT2_HETGA	118
40S ribosomal protein S27a	G5B8W4_HETGA	116
Inter-alpha-trypsin inhibitor heavy chain H2	G5AXV8_HETGA	106
Alpha-2-antiplasmin	G5BCV1_HETGA	86
Complement factor B	G5C3H1_HETGA	81
Elongation factor 1-alpha	G5ALK7_HETGA	78
Angiotensinogen	G5BQI5_HETGA	77
Histone H2B	G5BH20_HETGA	77
Plasma kallikrein	G5BNV2_HETGA	72
Beta-enolase	G5BW96_HETGA	70
Vitamin D-binding protein	G5BE53_HETGA	57
Alpha-1-antichymotrypsin	G5B491_HETGA	55
Keratin, type II cytoskeletal 80	G5BL95_HETGA	53
Heat shock cognate 71 kDa protein	G5B170_HETGA	51
Leucine-rich repeat-containing protein KIAA1731	G5C3Y1_HETGA	50
Cathepsin D	G5C2G1_HETGA	49
Basement membrane-specific heparan sulfate proteoglycan core protein	G5BI06_HETGA	47
Skin-specific protein 32	G5BUY9_HETGA	46
Desmocollin-1	G5C312_HETGA	46
Protein-arginine deiminase type-6	G5BZN1_HETGA	46
Cryptochrome-1	G5C454_HETGA	45
Complement C1q subcomponent subunit C	G5BHZ7_HETGA	45
p2Y purinoceptor 12	G5C102_HETGA	44
Alpha-1B-glycoprotein	G5B7K8_HETGA	44
Exportin-1	G5C1Y9_HETGA	44
Putative hexokinase HKDC1	G5B183_HETGA	43
Transcriptional regulator ATRX	G5C0I5_HETGA	42
Coiled-coil domain-containing protein 57	G5BJ98_HETGA	42
Microtubule plus-end tracking protein TIP150	G5ATU1_HETGA	40
Tubulin alpha-1C chain	G5AQ00_HETGA	38
Fer3-like protein	G5BZJ1_HETGA	38
Amino acid transporter	G5BYQ9_HETGA	36
Ventricular zone-expressed PH domain-containing protein-like protein 1	G5B253_HETGA	35
SRRM2-like protein	G5BG61_HETGA	35
N6-adenosine-methyltransferase 70 kDa subunit	G5BFU9_HETGA	33
Proline-rich protein 11	G5AXY5_HETGA	33
Carbonic anhydrase 2	G5ATW7_HETGA	32
26S proteasome non-ATPase regulatory subunit 3	G5BRY1_HETGA	31
Serine/threonine-protein kinase LATS1	G5BMD1_HETGA	31
Transmembrane gamma-carboxyglutamic acid protein 1	G5C418_HETGA	31
Collagen alpha-2(I) chain	G5ANK8_HETGA	31
Bcl-2-associated transcription factor 1	G5C1A7_HETGA	30

^#^ Ions score is −10 * Log(*p*), where *p* is the probability that the observed match is a random event. Individual ions scores > 30 indicated identity or extensive homology (*p* < 0.05). Protein scores were derived from ions scores as a non-probabilistic basis for ranking protein hits. Cut-off was set at Ions score 30. Protein hits identified in whole plasma only (but not in plasma EVs) are highlighted in blue.

## Supplementary Materials

Supplementary materials can be found at https://www.mdpi.com/1422-0067/20/21/5378/s1. Supplementary Table S1. Deiminated proteins identified by F95 enrichment in total plasma of naked mole-rat (*Heterocephalus glaber*). Deiminated proteins were isolated by immunoprecipitation using the pan-deimination F95 antibody. The F95 enriched eluate was analysed by LC-MS/MS and peak list files were submitted to mascot. Peptide sequences for the protein hits, their m/z values and individual scores are listed. Supplementary Table S2. Deiminated proteins identified by F95 enrichment in plasma-EVs of naked mole-rat (*Heterocephalus glaber*). Deiminated proteins were isolated by immunoprecipitation using the pan-deimination F95 antibody. The F95 enriched eluate was analysed by LC-MS/MS and peak list files were submitted to mascot. Peptide sequences for the protein hits, their m/z values and individual scores are listed.

## Abbreviations

BSA	Bovine Serum Albumin
CD63	CD63 antigen; granulophysin; lysosomal-associated membrane protein 3
CNS	Central Nervous System
ECL	Enhanced Chemiluminescence
EVs	Extracellular Vesicles
F95	Pan-deimination/citrullination antibody
FBS	Foetal Bovine Serum
Flot-1	Flotillin-1
HIF	Hypoxia-inducible Factor
KEGG	Kyoto Encyclopedia of Genes and Genomes
kDa	Kilodalton
LC-MS/MS	Liquid Chromatography Mass Spectrometry
miR	microRNA
NTA	Nanoparticle Tracking Analysis
PAD	Peptidylarginine Deiminase
SDS-PAGE	Sodium Dodecyl Sulfate Polyacrylamide Gel Electrophoresis
TBS	Tris Buffered Saline
TEM	Transmission Electron Microscopy
WB	Western Blotting
